# Exploring Loneliness, Trust, and Psychosocial Meanings Among Primary Care Attendees: A Cross-Sectional Study From a General Practice Setting in Crete, Greece

**DOI:** 10.7759/cureus.61019

**Published:** 2024-05-24

**Authors:** Panagiotis Volkos, Manolis Linardakis, Maria Stamati, Emmanouil K Symvoulakis

**Affiliations:** 1 Clinic of Social and Family Medicine, School of Medicine, University of Crete, Heraklion, GRC; 2 Department of Social Medicine, School of Medicine, University of Crete, Heraklion, GRC; 3 4th Local Health Team, Academic Unit of Heraklion, Heraklion, GRC

**Keywords:** social contacts, primary care, behavioral risk factors, trust, loneliness

## Abstract

Introduction: Loneliness is often associated with behavioral, psychological, social, and mental and physical health aspects, while the relationship between trust and loneliness is still challenging in terms of research. The present study aimed to investigate to what extent loneliness and trust interact and if there is any association between loneliness and routine events or behaviors that affect overall well-being.

Methods: This cross-sectional sample of 120 participants, aged between 40 and 75 years, was collected from an urban Primary Health Care Unit, between May and July 2023, in a consecutive manner, after criteria application. The University of California, Los Angeles (UCLA) Loneliness Scale (Version 3) and the Personal Trust and Connections Scale (PerTC) were completed. Hierarchical multiple linear regression analysis in steps and multiple logistic regression analysis were performed.

Results: The mean age of the participants was 59.8 years and the majority were females (73.3%). Only 10.8% were found to have no Behavioral Risk Factors (BRFs). A higher sense of loneliness was associated with being widowed/unmarried/divorced (unstandardized β=-4.10, p=0.045), not having children (β=-2.80, p=0.038), having more BRFs (β=2.03, p=0.032), or being in contact with fewer friends (β=-0.56, p<0.001). A higher sense of loneliness was related to lower levels of trust (β=-1.29, p=0.032). Lower odds of demonstrating high levels of loneliness were found among married individuals (odds ratio (OR): 0.38; 95% confidence interval (CI) 0.15, 0.95; p=0.039) and those who reported six or more routine contacts or meetings with friends (OR: 0.31; 95% CI 0.13, 0.74; p=0.008). A lower score on the total PerTC scale was related to higher odds of increased loneliness (OR: 2.78; 95%CI 1.01, 7.62; p=0.048).

Conclusion: Being married and coming in contact or meeting with more friends was associated with lower odds of reporting high feelings of loneliness, while lower trust was correlated with increased odds of loneliness. Also, persons with more BRFs were found to have a higher loneliness score. Given their inverse correlation, tools that measure levels of loneliness and trust, like the UCLA and PerTC scales, may be used within primary care consultation to offer new insights on health risk behaviors and their possible synergistic influences.

## Introduction

Loneliness is acknowledged as an unpleasant feeling that derives from objectively being or subjectively perceiving to be alone or isolated [1]. Feelings of loneliness are often considered to be important to citizens’ health, since people may often adopt harmful behaviors like smoking [2], physical inactivity [3], alcohol or substance use [4], and sugar intake [5], or even report disturbed sleep [6]. People may not perceive their feelings of loneliness as a co-variant of psychosocial harm. The term ‘psychosocial’ is used to describe the “meeting point” and the interplay between social, cultural, and environmental effects on the mind and behavior [7]. Moreover, loneliness can be associated with mental health complaints, such as depressive symptoms [8,9], several psychotic disorders [10], and psychological distress [11], while it is referred to as a mediator between emotional dysregulation and eating disorders like bulimia nervosa [12]. Therefore, the behavioral, psychological, social, and mental and physical health aspects of loneliness need to be taken into account.

More specifically, loneliness is related to a higher prevalence of being a smoker and a lower prevalence of quitting smoking in older adults [13], while those who start smoking present higher levels of loneliness [14]. A similar relationship between smoking and loneliness is reported among young adults [15]. A review showed that, of the studies that found a significant association between smoking and loneliness, only one found a negative association, while the rest of the studies reported that lonely people have a higher likelihood of being smokers [2].

Social isolation is associated with physical inactivity and a sedentary lifestyle in older people, although no associations with loneliness are found [16]. Increased weight is related to higher levels of loneliness between the ages of 12 and 13 years [17]. Obesity is positively associated with loneliness [18], while another study mentioned that obese men present lower loneliness while obese women have higher loneliness levels [19].

Moreover, higher levels of alcohol consumption were associated with loneliness for those reporting drinking more than twice per week but also for those reporting drinking less than once per week during the COVID-19 pandemic [20]. Additionally, a survey that took place prior to the pandemic found that people with moderate or severe loneliness drink alcohol more frequently compared to those with no feelings of loneliness [21]. Furthermore, participants with a low-risk drinking habit but high loneliness were more likely to develop a high-risk drinking attitude over time [22]. Multiple behavioral risk factors deserve attention since they can synergistically affect health and health outcomes, as has been reported [23]. Therefore, it is evident that feelings of loneliness need to be assessed during medical sessions since they can be associated with several hazardous behaviors, otherwise causatively unexplained.

Moreover, trust may also be related to how people perceive their socializing potential since people who are unable to trust others tend to have a weakness in social interactions and consequently feel lonely. Trust is one of the basic characteristics of human societies [24]. It appears to play an important role in cooperation and reciprocity in both social and economic interactions [25]. It is influenced by both biological and environmental factors such as culture and socialization [26]. It has been reported that trust and loneliness are negatively correlated [27]. Interpersonal trust acts as a mediating factor in the relationship between markers of inflammation and the existence of psychological trauma [28]. Apart from interpersonal trust, institutional trust is also considered in medical research, since it may be related to public health outcomes. For instance, trust in governments and health workers was found to be related to higher levels of positive views regarding vaccination and compliance with medical advice [29]. Exceptionally low levels of trust may lead to low adherence to medical treatment or late medical care seeking [30]. Therefore, trust, either interpersonal or institutional, within the health sector must be taken into account when investigating health behaviors and attitudes.

Rather than that, the importance of studying about loneliness, especially among primary care users, is due to reports that describe loneliness and social isolation as significant concerns for public health [31,32], while it has been proposed that social connection might help in reducing the effects of loneliness in the community [33]. Thus, it could be suggested that trust may play a key role in the way people shape or promote their social connections. Also, according to a study carried out in the United States, socioeconomic determinants and health behaviors contribute to 47% and 34% of health outcomes, respectively [34]. Due to the scarce evidence on studying those associations combined, it is believed that to perform such research may be essential at local and international levels.

Given the above, it is clear that loneliness and trust need to be studied together and in combination with other known behavioral factors that can affect overall health. The main questions that led to the present study concerned: (1) How loneliness and trust, both measured by validated research tools, may interact among persons who seek primary care services? and (2) How much is loneliness linked with routine events or behaviors driven by psychosocial determinants and affects overall well-being? As it was mentioned above, the motivation for answering those questions lies firstly in the evidence identifying loneliness as a public health issue that could affect health [32], with pleiotropic mechanisms. Also, it may be imperative for researchers and medical professionals if the aforementioned associations could be established by using simple but reliable research tools. Those tools could be used in order to early detect the establishment of a harming behavior and assess its impact on co-morbidity or other risk factors. The present study aimed to investigate to what extent loneliness and trust interact and if there is any association between loneliness and routine events or behaviors that affect overall well-being.

## Materials and methods

The present study was a primary healthcare-based observational and cross-sectional survey. The study setting was the 4th TOMY (Topiki Monada Ygeias - Local Health Unit) of Heraklion, Crete, Greece, which is an urban Primary Health Care (PHC) unit and the study population consisted of the registered adults of the PHC unit, between 40 and 75 years of age. In order to participate in the study, the following inclusion criteria had to be met: age between 40 and 75 years old, fluency in reading, writing, and comprehending the Greek language, and a Body Mass Index (BMI) ≤29.9 kg/m^2^. Additionally, specific exclusion criteria were applied. Individuals with major head trauma or major mental health disorders and those being pregnant during the study period or breastfeeding could not participate in the study. The collection of the sample took place between May and July 2023 and 120 participants were included from 288 scheduled appointments during two morning sessions weekly, in a consecutive manner, after the aforementioned criteria were applied.

Data collection

A semi-structured information sheet was designed and used to collect the required personal and health information for the present study. The semi-open questions mostly referred to morbidity type, medicines, and numeric responses. Researchers from different professional fields (medical, nursing, and social work) agreed and approved the reliability and clarity of the sheet before use. The information collected, included participants’ age (years), gender (male, female), marital status (unmarried, divorced, widowed, married), number of children in the family (number), level of education (primary school, junior high school, high school, technical education, university/technological school, MSc/PhD), occupational status (employed, unemployed, retired), height (cm), weight (kg), smoking habit (yes, no), alcohol consumption (yes, no; number of drinks per week), chronic diseases (list of reported diseases), prescribed medicine (list of reported medicine), any psychiatric diagnosis (yes, no), number of friends they routinely came in contact with, either face-to-face or online, during the last six months (number), individual/ group physical activity or sport during the last year (yes, no), experience of a dramatic event in the family during the last year (yes, no), duration of nocturnal sleep (hours), and current or former care of a pet (yes, no).

The subjective feeling of loneliness was assessed with the University of California, Los Angeles (UCLA) Loneliness Scale (Version 3) [35], validated in Greek by Pikea et al. (2016) [36]. The scale measures feelings of loneliness and social alienation and consists of 20 items (11 expressed negatively and nine expressed positively). Responses were given based on a 4-point Likert-type scale: never=1, rarely=2, sometimes=3, and always=4. In order to assess the final score, the nine items are reversed (questions 1,5,6,9,10,15,16,19,20) and then added up, resulting in a total score with a range of 20-80. A higher score indicates a greater subjective feeling of loneliness. The following cut-offs were adopted: total score <28 as no or low feeling of loneliness, 28 to 43 as moderate feeling, and >43 as high feeling [37,38]. Its reliability in the 120 patients was assessed with Cronbach’s α=0.880.

The Personal Trust and Connections Scale (PerTC) measures a person’s tendency to seek trustworthy connections in their daily life [39]. It includes 10 questions (items), in a graded, closed-type Likert scale (0: not at all to 10: very much). Three subscales or components comprise the scale as follows: (1) Emotional Reliance related to feelings of trust (questions 1,2,6,9,10), (2) Social Reliance related to social connections (questions 3,5,8), and (3) Cognitive Reliance related to elements that shape rational concepts of trust (questions 4 and 7, with question four reversed due to opposite conceptual content). The total scale score and that of its three subscales were measured by estimating the average value of the answers to the corresponding questions of each subscale as well as to the total of 10 questions, creating scores of a similar and comparable range. PerTC was originally developed and validated within Greek respondents by members of the current research team [39]. The adopted cut-off of 6.7 (<6.7 vs. 6.7+) corresponds to 2/3 (67%) of the score distribution. The total scale reliability in the 120 patients was assessed with Cronbach’s α=0.734.

Behavioral Risk Factors (BRFs) for chronic diseases were defined as: (1) increased body weight according to BMI (overweight or ≥25 kg/m^2^ compared to normal or <25 kg/m^2^), (2) lack of physical activity (lack of scheduled, at least once weekly, individual/group physical exercise or sport during the last year), (3) smoking habit, and (4) any consumption of alcoholic beverages per week. After each factor received the value one or '1' as presence, they were aggregated, defining the Behavioral Risk Factors as the absence and the combined presence of the four behaviors (composite score, from 0: no factor to 4: all four) [23].

Insufficient sleep duration per night was defined as <6 hours [40]. The consumption of any alcoholic beverage per week was adopted since, according to the World Health Organization (WHO), there is no standard level of alcohol drinking that can be considered safe [41].

Ethics

Approval to conduct this study for the needs of an ongoing PhD thesis was obtained from the Ethics and Deontology Committee of the University of Crete (ref. no. 166/11.11.2022). The research protocol was also approved by the 7th Health Regional Authority of Crete (ref. no. 6460) and the study was performed in accordance with the Declaration of Helsinki. Written informed consent was obtained from the participants.

Statistical analysis

Data analysis was carried out using the SPSS software, version 25.0 (IBM Corp., Armonk, NY). Frequency distributions and measures of location and dispersion of the characteristics of the 120 participating primary care attendees were calculated. The distributions of the scores of the UCLA and PerTC scales were checked using Blom’s method (QQ plot) and the three PerTC subscales (Social, Cognitive, and Emotional) were compared using analysis of variance. The internal consistency of responses of the two scales was assessed using Cronbach’s α coefficient. The correlation of the levels of the UCLA Loneliness Scale with socio-demographic characteristics (1st step), health habits and characteristics (2nd step), and the PerTC Scale (3rd and 4th steps) was checked using nested or hierarchical multiple linear regression analysis in steps. The structure of the models involved the following steps and factors the 1st step was determined by an analysis model (multiple linear regression) involving the main socio-demographic characteristics (gender, age, family status, children, education level, and occupation); the 2nd step was determined by the 1st step and health habits and characteristics such as BRFs, sleeping hours at night, chronic diseases, routine contacts or meetings with friends during the last six months, current or former care of a pet, and recent dramatic event in family during the last year (two hierarchical models); the 3rd step was determined by the 1st and 2nd steps and the three PerTC components, Social, Cognitive, and Emotional reliance (three hierarchical models); the 4th step was determined by the 1st and 2nd steps and the total PerTC scale (also three hierarchical models). Moreover, multiple logistic regression analysis of higher levels of loneliness (UCLA Loneliness Scale) in relation to socio-demographic characteristics (gender, age, family status, children, education level, and occupation), health habits and characteristics (sleeping hours at night, routine contacts or meetings with friends during the last six months, and recent dramatic event in family during the last year), and trust (PerTC scale) was implemented. The acceptable level of significance was set at p<0.05.

## Results

Most participants were women (n=88; 73.33%), while the mean age of the sample was 59.8 years (±9.5). Eighty-one (67.5%) participants were married and 85.8% (n=103) had at least one child. Twenty-five percent (n=30) said they had university-level education, 57.5% (n=69) were unemployed or retired, and the majority (114, 95.0%) were insured (Table 1).

**Table 1:** Characteristics of study participants All 120 study participants were primary care unit attendees.

Socio-demographic characteristics		n	%
Gender	male/female	32 / 88	26.7 / 73.3
Age, years	mean±stand. dev.	59.8±9.5	
Family status	unmarried	9	7.5
	divorced	14	11.7
	widowed	16	13.3
	married	81	67.5
Children	none	17	14.2
	1	11	9.2
	2+	92	76.6
Education level	Up to junior high school	37	30.8
	High school	34	28.3
	Technical education	19	15.9
	University/Technological School MSc, PhD	30	25.0
Occupation	employed	51	42.5
	unemployed, retired	69	57.5
Health Insurance	yes	114	95.0

According to Table 2, 79 (65.8%) participants were found to be overweight according to BMI (overweight: ≥25 kg/m^2 ^and ≤29.9 kg/m^2^, compared to normal: ≥18.5 kg/m^2^ and <25 kg/m^2^), 68 (56.7%) had no physical activity during the last year, 36 (30%) were active smokers, and 36 (30%) mentioned consuming at least one drink during the week. Only 10.8% (n=13) of the participants had no BRFs. Mean hours of nocturnal sleep were calculated at 6.7 hours (±1.2). Moreover, 104 (86.7%) participants had a chronic disease, while the median number of persons, reported as friends, that every participant routinely came in contact with or met in the last six months was six (interquartile range (IQR): 4, 10). Additionally, 62 participants (51.7%) mentioned taking or formerly taking care of a pet, and 44 (36.7%) reported experiencing a dramatic event in their family during the last year.

**Table 2:** Health habits and characteristics of the 120 primary care unit attendees (study participants) ^a^Some participants displayed co-morbidity.

Health habits and characteristics		n	%
Increased body weight	overweight/normal	79 / 41	65.8 / 34.2
Lack of physical activity	yes/no	68 / 52	56.7 / 43.3
Smoking	yes/no	36 / 84	30.0 / 70.0
Alcohol consumption	yes/no	36 / 84	30.0 / 70.0
Behavioral Risk Factors (Presence of four behaviors as composite score 0-4)	none	13	10.8
	1	29	24.2
	2	47	39.2
	3	28	23.3
	4	3	2.5
Sleep hours at night	mean±stand. dev.	6.7±1.2	
	<6 hours	16	13.3
Chronic diseases^a^	no	16	13.3
	yes	104	86.7
	mental disorder	45	37.5
	hypothyroidism	21	17.5
Routinely contacting or meeting with friends during the last six months	median (IQR)	6 (4, 10)	
Current or former care of a pet	yes	62	51.7
Recent dramatic events in the family during the last year	yes	44	36.7

The sample had a UCLA scale mean score of 41.0 (±10.1), and most participants scored between 28.0 and 43.0 (moderate sense of loneliness) (n=63; 52.5%), as we see in Table 3. Moreover, the total PerTC mean score was 5.6 (±1.5), while the highest mean scores were observed in Social and Cognitive Reliance (mean=6.7; ±2.2; and mean=6.0; ±1.9, respectively), while the Emotional Reliance mean score was the lowest (4.9; ±1.8). The analysis of variance between the three components of PerTC was estimated at p<0.001.

**Table 3:** UCLA loneliness scale and the PerTC scale scores of the 120 study participants UCLA: University of California, Los Angeles; PerTC: Personal Trust and Connections ^a^Higher score (➡80) shows a higher sense of loneliness. ^b^Higher score (➡10) shows higher trust and connections; ^c^Analysis of variance between the three components of PerTC (F=25.1, d.f.=2, p<0.001). Homogeneity was tested by Levene’s test (Levene statistic=2.94, d.f.1=2, d.f.2=357, p=0.054).

Scales	n	mean	stand. dev.	median	min	max
UCLA Loneliness Scale ^a^	120	41.0	10.1	39.5	21	69
Absence or low sense of loneliness (<28.0)	11	9.2%				
Moderate sense (28.0-43.0)	63	52.5%				
High sense (>43.0)	46	38.3%				
Total Personal Trust and Connections scale (PerTC) ^b^	120	5.6	1.5	5.9	1.6	8.5
Social Reliance^c^	120	6.7	2.2	7.0	0.0	10.0
Cognitive Reliance^c^	120	6.0	1.9	5.8	0.0	10.0
Emotional Reliance^c^	120	4.9	1.8	5.0	0.0	9.0

**Figure 1:**
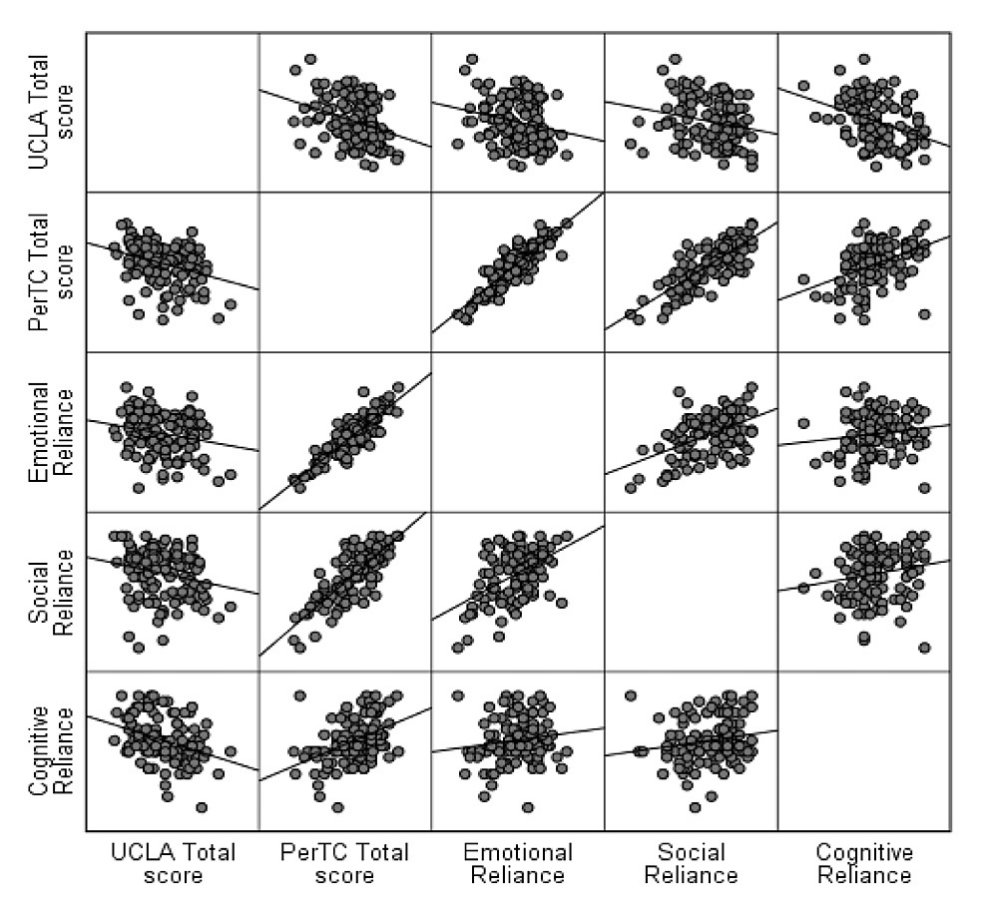
Score levels of the PerTC scale of the 120 study participants in accordance with the UCLA loneliness scale classification PerTC: Personal Trust and Connections; UCLA: University of California, Los Angeles

The relationship between the UCLA and PerTC scales in Figure 1 shows that the UCLA and PerTC scales, in total, are inversely correlated. A similar pattern was observed between UCLA and all three components of the PerTC scale. All correlation coefficients were significantly negative (p<0.05).

Table 4 presents the hierarchical multiple linear regression analysis of the levels of the subjective feeling of loneliness on the UCLA scale with socio-demographic characteristics (1st step), health habits and characteristics (2nd step), and the PerTC scale (3rd and 4th steps) of the study participants. The correlation of socio-demographic characteristics with the levels of loneliness (1st step) showed that widowed/unmarried/divorced people (unstandardized β=-4.10, p=0.045) or those without children (β=-2.80, p=0.038) scored higher on the UCLA scale. By adding the factors of the 2nd step (health habits and characteristics along with socio-demographic characteristics), those with more BRFs (β=2.03, p=0.032) or those who routinely came in contact with or met with fewer friends during the last semester were found to have a significantly higher score or feeling of loneliness (β=-0.56, p<0.001). Additionally, when the three components of the PerTC scale were added together (3rd step), those with significantly higher scores or feelings of loneliness presented lower levels of Cognitive reliance (β=-1.18, p=0.012). Finally, when the overall PerTC scale was added (4th step), significantly higher scores or feelings of loneliness were detected in those with lower levels of PerTC (β=-1.29, p=0.032).

**Table 4:** Hierarchical multiple linear regression analysis This table presents the hierarchical multiple linear regression analysis of the levels of the University of California, Los Angeles (UCLA) Loneliness Scale with socio-demographic characteristics (1st step), health habits and characteristics (2nd step), and the Personal Trust and Connections (PerTC) scale (3rd and 4th steps) of the 120 study participants. Model factors: the 1st step was determined by an analysis model (multiple linear regression), the main socio-demographic characteristics (gender, age, etc.); the 2nd step was determined by the 1st step and health habits and characteristics such as Behavioral Risk Factors, sleeping hours, chronic diseases, etc. (two hierarchical models); the 3rd step was determined by the 1st and 2nd steps and the three PerTC components, Social, Cognitive and Emotional reliance (three hierarchical models); the 4th step was determined by the 1st and 2nd steps and the Total Personal Trust and Connections scale (also three hierarchical models). Bold indicates significant unstandardized β coefficients.

Particulars		UCLA Loneliness Scale		
Steps	Prognostic factors	unstandardized coefficient β	standard error	p-value
1^st^	Gender (1:male, 2:female)	-1.42	2.04	0.486
	Age (years)	0.13	0.12	0.290
	Family status (1: unmarried, divorced, widowed, 2: married)	-4.10	2.02	0.045
	Children (1: none, 2:one, 3:two or more)	-2.80	1.34	0.038
	Education level (1:up to junior high school, 2:high school, 3:Technical education, 4: University/Technological School, MSc, PhD)	-0.22	0.82	0.792
	Occupation (1:employed, 2:unemployed, retired)	-2.68	2.16	0.218
	R^2^(adj.)	0.071		
2^nd^	Behavioral Risk Factors (sum of composite score 0-4)	2.03	0.93	0.032
	Sleep hours at night (1:<6 hours, 2:6+ hours)	3.19	2.50	0.204
	Chronic diseases (sum)	0.38	2.81	0.892
	Routine contacts or meetings with friends during the last 6 months (sum)	-0.56	0.15	<0.001
	Current or former care of a pet (1:no, 2:yes)	3.35	1.77	0.061
	Recent dramatic events in family during the last year (1:no, 2:yes)	3.33	1.78	0.064
	R^2^(adj.)	0.230		
3^rd^	Social Reliance	-0.29	0.46	0.530
	Cognitive Reliance	-1.18	0.45	0.012
	Emotional Reliance	-0.32	0.54	0.555
	R^2^(adj.)	0.274		
4^th^	Total Personal Trust and Connections scale	-1.29	0.60	0.032
	R^2^(adj.)	0.256		

According to the multiple logistic regression analysis, significantly lower odds ratios (adjusted) of demonstrating high feelings of loneliness were detected among married individuals compared to unmarried, divorced, and widowed counterparts (odds ratio (OR): 0.38; 95% confidence interval (CI) 0.15, 0.95; p=0.039) and among those who routinely came in contact with or met six or more friends during the last six months compared to those who reported up to five routine contacts or meetings with friends (OR: 0.31; 95% CI 0.13, 0.74; p=0.008) (Table 5). Concerning total PerTC scores, higher odds ratios (adjusted) of increased levels of loneliness were associated with lower scores on the total PerTC scale (OR: 2.78; 95% CI 1.01, 7.62; p=0.048).

**Table 5:** Multiple logistic regression analysis This table presents the multiple logistic regression analysis of higher levels of loneliness (University of California Los Angeles (UCLA) Loneliness Scale) in relation to socio-demographic features, health habits, social and family events, and trust (Personal Trust and Connections (PerTC) scale) of the 120 study participants. ^a ^The cut-off of 6.7 corresponds to 2/3 (67%) of the score distribution.

Prognostic factors	University of California Los Angeles Loneliness Scale (UCLA) (High Sense of Loneliness vs. Moderate/Low Sense)		
	odds ratio, OR (adjusted)	95%CIs	p-value
Gender (female vs. male)	0.70	0.27, 1.81	0.462
Age (per 5-year change)	1.14	0.83, 1.57	0.408
Family status (married vs. unmarried, divorced, widow)	0.38	0.15, 0.95	0.039
Children (yes vs. no)	0.34	0.10, 1.18	0.089
Education (for each level of increase)	1.02	0.69, 1.51	0.929
Occupation (unemployed, retired vs. employed)	0.33	0.12, 1.07	0.066
Sleep hours at night (<6 hours vs. 6+ hours)	2.72	0.81, 9.19	0.106
Routine contacts or meetings with friends during the last 6 months (6+ persons vs. 0-5 persons)	0.31	0.13, 0.74	0.008
Recent dramatic events in family during the last year (yes vs. no)	1.85	0.74, 4.63	0.188
Total Personal Trust and Connections scale (<6.7 vs. 6.7+) ^a^	2.78	1.01, 7.62	0.048
R^2^ Nagelkerke	0.289		

## Discussion

The present study found higher levels of perceived loneliness among unmarried, divorced, or widowed individuals, those without children, and those who routinely came in contact with fewer friends during the last semester. Moreover, participants who mentioned having more BRFs (smoking, higher BMI, alcohol consumption, and physical inactivity) scored higher on the UCLA scale. Nevertheless, comparing the UCLA scale with the PerTC scale, it was found that higher levels of loneliness were associated with a lower total PerTC score and a lower score in Cognitive reliance on the same scale. Furthermore, significantly lower odds of reporting high feelings of loneliness were found among married individuals and those who routinely came in contact with or met six or more friends during the last semester, while higher odds of increased loneliness levels were observed between those with lower scores in total PerTC scale.

The current results regarding loneliness and marital status were consistent with a systematic review which reported that people without a partner or those who had lost a partner were at a high risk of feeling lonely [42], while being divorced or widowed was also associated with increased odds of loneliness [11]. The same study mentioned that increased risk of loneliness was detected among people with a limited social network [42], which is similar to the present finding regarding people who routinely come into contact with fewer friends. Therefore, it may be argued that subjective and objective loneliness are connected. Objective time spent alone and feelings of loneliness have been reported to be positively interrelated [43], while low levels of social relationships are associated with increased odds of stroke and coronary heart disease incidents [44], indicating that loneliness affects physical health. Social isolation, loneliness, and living alone also showed a high likelihood of mortality, 29%, 26%, and 32% respectively [45]. These terms seem to be connected and highlight the complexity of loneliness as a feeling, and the importance of acting in order to buffer or prevent it.

Concerning the relation between loneliness and trust, the study by Lieberz et al. (2021) found that participants with higher loneliness, measured by UCLA, exhibited less trust towards others, both during a trust game and in functional magnetic resonance imaging (MRI) findings [46]. The present study assessed trust by using a scale, finding a negative association with loneliness as indicated by the hierarchical multiple linear regression analysis. Moreover, the Cognitive reliance of the PerTC scale was also negatively associated with the loneliness scale, probably indicating the cognitive aspect of subjective loneliness. Peplau and Caldwell (1978) earlier gave a cognitive dimension to loneliness by exploring the process of how people classify themselves as “lonely” and the source of their loneliness [47]. There are also studies connecting loneliness to cognitive decline in older individuals [48]. Further research is needed in order to explore the cognitive features of loneliness in detail.

According to the present data, loneliness affects people’s behavior, specifically behaviors that may be involved in the deterioration of physical health, such as smoking, drinking, lack of physical activity, and increased body weight. There is evidence that loneliness is associated with smoking or lower possibilities of quitting smoking, especially when loneliness co-exists with self-reported emotional problems [13], while lack of close friends and loneliness have also been related to smoking [49]. Studying behavioral risk factors is also challenging, although their separate effects on chronic disease occurrence [23,50] and all-cause mortality [51] are already known. For instance, smoking and physical inactivity contribute to both cardiovascular disease (CVD) and total mortality [52]. Additionally, loneliness has been negatively correlated with self-care ability (including, among others, physical, psychological, and emotional components) [53], indicating that interventions to reduce loneliness levels could be beneficial in several aspects of someone’s life.

Based on our findings, it may be argued that both cognitive and behavioral aspects can be found within subjective feelings of loneliness that may eventually influence people’s overall health. This further proof of the relationship between health and cognitive-behavioral aspects supports the implementation of preventative measures. Moreover, early signs of high levels of feelings of loneliness or lower levels of trust could be taken into account, in a joint manner, by policymakers or stakeholders when designing individual or community health planning and interventions. Additionally, the early detection of non-typical and hidden risk factors, but potentially able to be intercepted, could help health professionals to manage harming health behaviors for patients who attend their settings, with an apparently different reason for encounter. The UCLA and PerTC scales are recommended as easy-to-use tools. Primary care professionals can play a crucial role in identifying, referring, or intervening in such cases since the PHC environment allows professionals to obtain a thorough medical history, with many behavioral elements. Last but not least, they could be involved in the design and implementation of community interventions regarding building trustful relations and offering motivation.

Strengths and limitations

One of the strengths of this study was that it demonstrated a clear link between loneliness and trust and BRFs. Loneliness is undoubtedly associated with some of the presented BRFs. The authors attempted to use a transatlantic, valid metric tool in conjunction with a locally designed scale to extrapolate local primary care data. Moreover, the study established an inverse correlation between the newly developed PerTC scale and the established UCLA Loneliness scale, aligning concepts and metrics.

Furthermore, Greece, without any intention to dramatize, met a major financial crisis with much uncertainty for years, and subsequently, this national adventure lived also the global sanitary crisis of COVID-19. Due to the fact that income has been noted in the literature to play a key role in the sense of loneliness [54,55], researchers find extremely important the study of health and social determinants and the ideal environment, perhaps, is primary care since consultations cover a range of issues in terms of comprehensiveness and motivational understanding.

One of the main limitations of the study is due to its design, which was cross-sectional. Therefore, causal association cannot be deduced, and from one setting. Thus, extended conclusions should be treated with care. Furthermore, the sample size does not allow for oversimplification of the results. However, the present study did not intend to generalize these findings to the entire primary care population. Participants also had to recall several occasions that may have occurred during the last six months or year (communication with friends, incidence of dramatic events), so recall biases cannot be excluded.

Revisiting with a backward manner the study design, the authors believed that was proved appropriate. Additionally, the use of specific criteria, such as age (40-75) and BMI (≤ 29.9/m^2^), supported the acquisition of lower uncertainty regarding the studied correlations. For instance, younger generations are differently affected by loneliness [56]. Rather than that, the way that obesity interplays with loneliness and social isolation is still under research [57]. Therefore, the levels of loneliness observed show some clear correlations with trust levels and routine contact with friends and family. Marital status [58], number of friends [59] and trust levels [27,60] have all been reported to be somehow related to loneliness.

This study targeted overweight attendees (with BMI ≤ 29.9/m^2^) to prevent confounding interaction between obesity and the eventuality of feeling lonely because of the previous [61]. According to a systematic review concerning the link between obesity and loneliness or social isolation, while some studies identified an association between obesity and augmented loneliness levels, it should be acknowledged that observations were still not clear [57]. Gender differences need further exploration as well.

## Conclusions

The present findings demonstrated a reverse correlation between loneliness and trust, both measured by instruments that are easily used in daily practice thanks to their non-time-consuming design and user-friendly content. Additionally, several health behaviors, such as increased body weight, lack of physical activity, smoking, and alcohol consumption, were found to be related to loneliness. Also, lower odds of having high feelings of loneliness were associated with family status (married) and those who routinely came in contact with or met more friends. These findings show that loneliness is a key factor that needs to be explored, identified, and handled by healthcare or mental health professionals in order to promote overall physical and mental health. Given the fact that loneliness was found to be inversely associated with trust, both need to be studied simultaneously and with equal emphasis. A primary healthcare environment could be suitable to include inclusive strategies to intercept feelings of loneliness and enhance trustful relationships due to the continuity of tight care delivery between the primary care user and the practice team members.
